# Ocular Ultrasound Elastography for Intraocular Tumors: A Pilot Study

**DOI:** 10.7759/cureus.106999

**Published:** 2026-04-13

**Authors:** Hector G Moreno Solano, Carlos Rios Elizondo, Gabriela E Ibarra Elizalde, Claudia M Cuevas Menéndez, David Ancona-Lezama

**Affiliations:** 1 Retina and Vitreous Surgery, Instituto Mexicano de Oftalmología I.A.P, Querétaro, MEX; 2 Ocular Ecography, Eye Cancer Institute, Monterrey, MEX; 3 Ocular Ecography, Centro de Alta Especialidad en Ultrasonido Ocular, Guadalajara, MEX; 4 General Ophthalmology, Asociación para evitar la ceguera en México, Ciudad de México, MEX; 5 Ocular Oncology, Eye Cancer Institute, Monterrey, MEX

**Keywords:** ocular manifestations of cancer, ocular oncology and medical retina, ocular tumor, ultrasonography (us), usg elastography

## Abstract

Ocular ultrasound elastography (OcUsE) was evaluated as a novel adjunct imaging modality for the characterization of intraocular tumors. In this prospective pilot study, 12 patients (12 eyes) with clinically and imaging-confirmed intraocular tumors underwent conventional B-scan ultrasonography complemented by strain and shear-wave elastography using a high-frequency linear probe. Quantitative tumor stiffness values, expressed in kilopascals, and qualitative elastographic patterns were recorded, correlated with multimodal imaging findings, and analyzed for diagnostic accuracy in differentiating benign from malignant lesions. Elastography was successfully performed in all cases without procedure-related complications.

Choroidal melanomas demonstrated high stiffness values (58.4 ± 12.1 kPa), choroidal hemangiomas showed low stiffness (18.9 ± 6.4 kPa), metastases exhibited heterogeneous stiffness profiles (32.6 ± 9.8 kPa), and retinoblastoma showed intermediate stiffness (41.2 kPa). A shear-wave elastography cutoff of 28 kPa achieved a sensitivity of 91.6% and a specificity of 100% for malignancy discrimination, with excellent interobserver agreement (intraclass correlation coefficient = 0.91). These findings indicate that OcUsE is a safe, feasible, and reproducible technique that provides quantitative biomechanical information and may enhance the differentiation of benign and malignant intraocular tumors, supporting further validation in larger prospective studies.

## Introduction

Intraocular tumors present significant diagnostic challenges, as conventional imaging modalities primarily provide structural information without direct assessment of tissue biomechanical properties [1,2]. Ocular ultrasound elastography (OcUsE), as outlined in the OcUsE protocol, is a novel adjunctive technique that generates quantitative stiffness measurements in conjunction with standard ultrasonography. The primary objective of this prospective pilot study was to assess the feasibility and diagnostic performance of OcUsE in differentiating benign from malignant intraocular tumors, while secondarily evaluating stiffness characterization and measurement reproducibility. By integrating biomechanical assessment with conventional imaging, this approach aims to address recognized limitations of structural imaging in ocular oncology.

The technique was successfully performed in all patients, with acquisition times comparable to standard B-scan ultrasonography and without procedure-related adverse events. These findings are consistent with the established safety profile of ultrasound elastography in other medical disciplines, including hepatology and soft tissue oncology, where elastography has been widely adopted as a non-invasive adjunct for lesion characterization and risk stratification [3-6].

Distinct elastographic stiffness profiles were observed across tumor subtypes, paralleling known histopathologic and structural characteristics. Choroidal melanomas consistently exhibited high stiffness values, likely reflecting their dense cellularity and compact stromal architecture. In contrast, choroidal metastases exhibited heterogeneous stiffness patterns, reflecting their variable cellular composition and infiltrative growth behavior. Choroidal hemangiomas showed significantly lower stiffness values, consistent with their vascular nature and loose tissue structure. The single retinoblastoma case exhibited intermediate stiffness with patchy heterogeneity, plausibly influenced by intratumoral calcifications, a hallmark feature on conventional imaging [7]. Although no prior ocular elastography series exist for direct comparison, these findings are concordant with elastography studies in other solid tumors, in which malignant lesions typically demonstrate higher stiffness than benign entities, reflecting underlying tumor biology and mechanobiology [8-11].

The identification of a shear-wave elastography cutoff value of 28 kPa with high sensitivity and specificity for malignancy discrimination is a clinically relevant observation. While this threshold requires validation in larger and more diverse populations, it suggests that elastography may aid in differentiating benign vascular tumors from malignant intraocular lesions, particularly in cases where conventional ultrasonography or MRI findings are inconclusive [12-14].

Correlation between elastographic findings and conventional imaging modalities further supports the potential clinical utility of OcUsE. The B-scan ultrasonography reliably confirmed tumor size and internal reflectivity; optical coherence tomography (OCT) delineated secondary retinal changes, and MRI, when available, was concordant with elastographic tumor margins. The MRI remains the reference standard for local tumor characterization and staging, particularly in retinoblastoma and selected uveal tumors [15-18].

## Materials and methods

A prospective, cross-sectional, experimental pilot study was conducted under the OcUsE protocol. Approval was obtained from the Eye Cancer Institute Institutional Review Board (approval no. ECI-IRB-2025-003). All procedures conformed to the tenets of the Declaration of Helsinki.

From January to June 2025, consecutive adult and pediatric patients referred to the Ocular Oncology Service with a clinical diagnosis of an intraocular tumor were enrolled. Inclusion criteria comprised: (1) the presence of an intraocular mass documented by clinical examination and conventional B-scan ultrasonography, (2) eligibility for ocular ultrasound with elastography, and (3) written informed consent provided by the patient or by a legal guardian for minors. Exclusion criteria were (1) media opacities precluding adequate ultrasound insonation, (2) a history of major ocular surgery within the preceding three months, and (3) refusal to participate.

**Figure 1:**
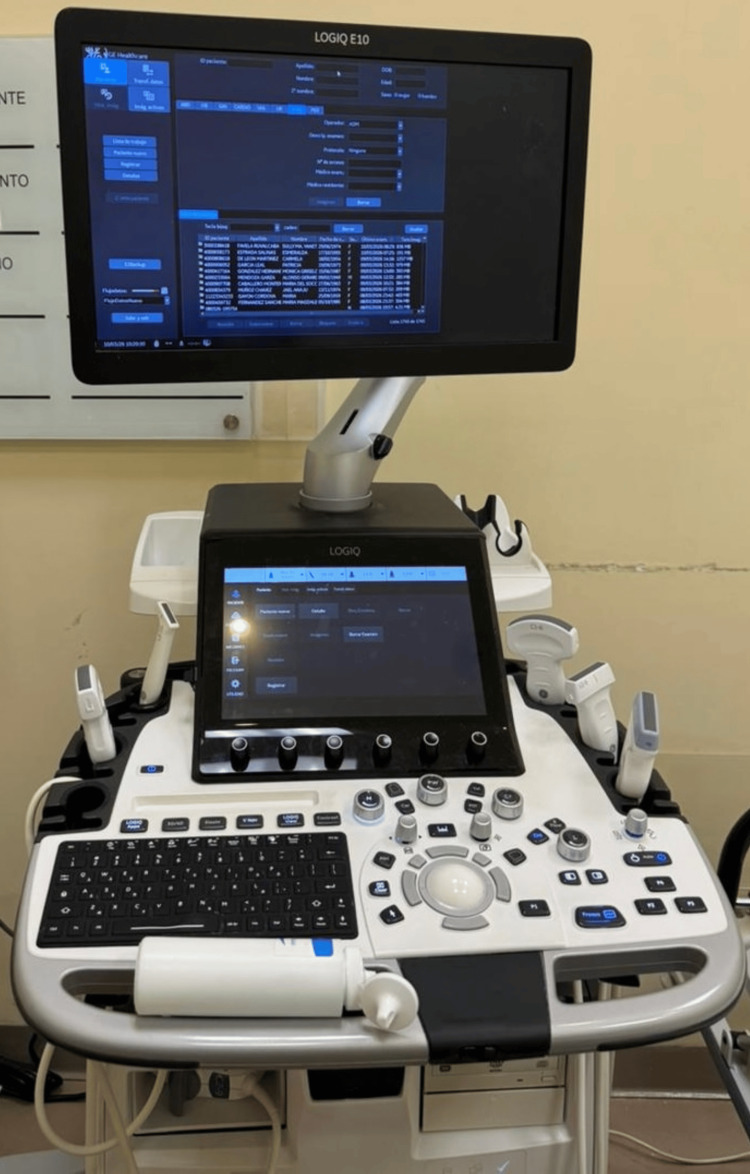
The LOGIQ E10 (GE Healthcare) used for ultrasound image acquisition

All patients underwent standard ocular ultrasonography using a high-frequency ultrasound system equipped with a 10-18 MHz linear probe to assess tumor dimensions, thickness, internal reflectivity, and acoustic characteristics, which remain the reference standard for intraocular tumor evaluation. Examinations were performed using a LOGIQ E10 ultrasound system with ElastQ shear-wave elastography (LOGIQ systems, GE Healthcare, Chalfont St. Giles, UK) adapted for high-resolution ocular imaging (Figure 1).

Following conventional B-scan imaging, examinations were complemented with shear-wave elastography. Quantitative stiffness measurements were obtained using standardized region-of-interest (ROI) placement within the solid portion of the tumor, avoiding areas of overt calcification or signal instability when feasible. Multiple measurements were acquired per lesion to ensure stability and reproducibility, and values were accepted only when adequate shear-wave propagation and minimal variability were confirmed according to system quality indicators.

Ultrasound examinations were performed with the patient in the supine position. The transducer was gently applied over the closed eyelid using sterile coupling gel as the acoustic interface, with particular care taken to avoid excessive pressure and compression artifacts. Each lesion was evaluated in multiple planes, including transverse and longitudinal orientations. Elastographic maps were generated to display color-coded tissue stiffness distribution, and quantitative elasticity values were obtained in kilopascals (kPa). For each tumor, minimum, maximum, and mean stiffness values were recorded. To minimize interobserver variability, all examinations were independently performed by two ophthalmologists experienced in ocular ultrasonography and elastography.

The primary outcome measure was the feasibility and diagnostic performance of shear-wave elastography in differentiating benign from malignant intraocular tumors. Diagnostic performance was explored through quantitative stiffness measurements and analysis of an optimal cutoff value for malignancy discrimination.

Secondary outcomes included characterization of mean tumor stiffness (kPa) across tumor subtypes, qualitative elastographic patterns (homogeneous versus heterogeneous stiffness distribution), interobserver and intraobserver reproducibility, acquisition time, and procedure-related adverse events. Correlation with the clinical presumptive diagnosis, i.e., uveal melanoma, intraocular metastasis, choroidal hemangioma, and retinoblastoma, was also evaluated.

Statistical analysis was performed using SPSS Statistics, version 27 (IBM Corp., Armonk, NY, USA). Data distribution was assessed with the Kolmogorov-Smirnov test. Comparisons between groups were conducted using Student’s t test or analysis of variance (ANOVA) for normally distributed variables and the Mann-Whitney U test for non-normally distributed variables. Interobserver agreement was quantified using the kappa statistics [19]. A two-tailed p-value of less than 0.05 was considered statistically significant.

## Results

A total of 12 patients (12 eyes) with intraocular tumors were enrolled. The mean age was 51.7 ± 16.3 years (range, 24 to 73 years), and seven (58.3%) were female. The cohort included six choroidal melanomas (50%), three choroidal (25%), two circumscribed choroidal hemangiomas (16.7%), and one retinoblastoma (8.3%) (Table 1).

**Table 1:** Demographic and baseline characteristics

Variable	Value
Age, mean ± SD (years)	51.7 ± 16.3
Sex: Male, n (%)	5 (41.7)
Sex: Female, n (%)	7 (58.3)
Choroidal melanoma, n (%)	6 (50.0)
Choroidal metastasis, n (%)	3 (25.0)
Choroidal hemangioma, n (%)	2 (16.7)
Retinoblastoma, n (%)	1 (8.3)

Feasibility, safety, and elastographic findings

**Figure 2:**
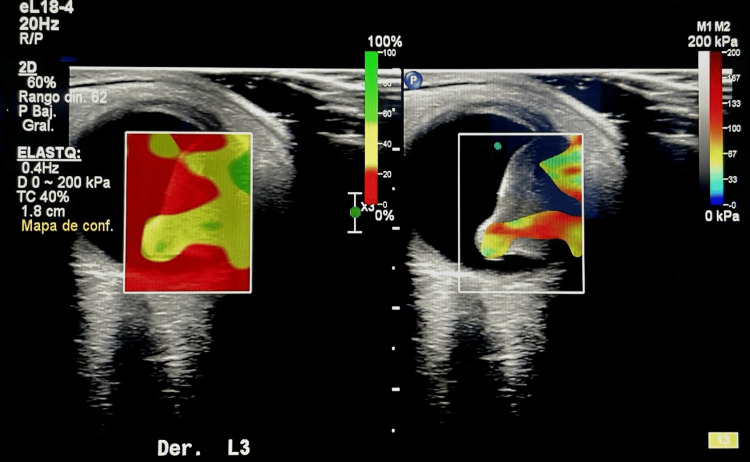
Choroidal melanoma elastography

Elastography was successfully performed in all 12 eyes (100%). Both strain and SWE produced interpretable stiffness maps, with no cases excluded due to poor image quality. The mean acquisition time was 6.5 ± 2.3 minutes per eye, comparable to standard B-scan ultrasound. No adverse events were recorded. Specifically, there were no cases of corneal epithelial defects, pain requiring additional anesthesia, or significant intraocular pressure elevation (>5 mmHg from baseline) immediately or 24 hours after the procedure. Distinct elastographic patterns were observed across the different intraocular tumor entities. Choroidal melanomas consistently demonstrated high tissue stiffness on shear-wave elastography, with a mean stiffness of 58.4 ± 12.1 kPa. These lesions characteristically displayed homogeneous blue color patterns on strain elastography, reflecting uniformly increased rigidity (Figure 2).

In contrast, choroidal metastases exhibited intermediate stiffness values (32.6 ± 9.8 kPa) with marked intralesional heterogeneity. Elastographic maps frequently showed mixed blue-red color patterns, consistent with variable tumor cellularity and structural composition. Choroidal hemangiomas showed low stiffness values on shear-wave elastography, with a mean of 18.9 ± 6.4 kPa, and predominantly red color-coding on strain elastography, in keeping with their highly vascular nature. The single case of retinoblastoma, diagnosed in a three-year-old child, demonstrated intermediate stiffness (41.2 kPa) with patchy elastographic heterogeneity. These heterogeneous areas corresponded to regions of calcification identified on conventional B-scan ultrasonography.

Correlation with conventional imaging modalities supported the elastographic findings. The B-scan ultrasonography confirmed tumor size and internal reflectivity in all cases. The OCT delineated overlying retinal alterations, particularly in cases of choroidal metastasis and hemangioma. In the subset of patients who underwent MRI, tumor margins and extent were concordant with elastographic findings in all five cases evaluated.

Reproducibility analysis demonstrated excellent interobserver agreement for shear-wave elastography measurements (intraclass correlation coefficient (ICC) = 0.91) and very high intraobserver repeatability (ICC = 0.95). Strain elastography color patterns were reproducible in 10 of 12 cases (83.3%), with variability predominantly observed among metastatic lesions (Table 2).

**Table 2:** Elastography findings by tumor type

Tumor type	Mean stiffness (kPa)	Elastographic pattern	p-value <0.05
Choroidal melanoma	58.4 ± 12.1	Homogeneous (blue)	< 0.01 vs benign
Choroidal metastasis	32.6 ± 9.8	Heterogeneous (mixed)	< 0.01 vs hemangioma
Choroidal hemangioma	18.9 ± 6.4	Low stiffness (red)	Reference group
Retinoblastoma	41.2	Patchy heterogeneity	< 0.01 vs benign

**Figure 3:**
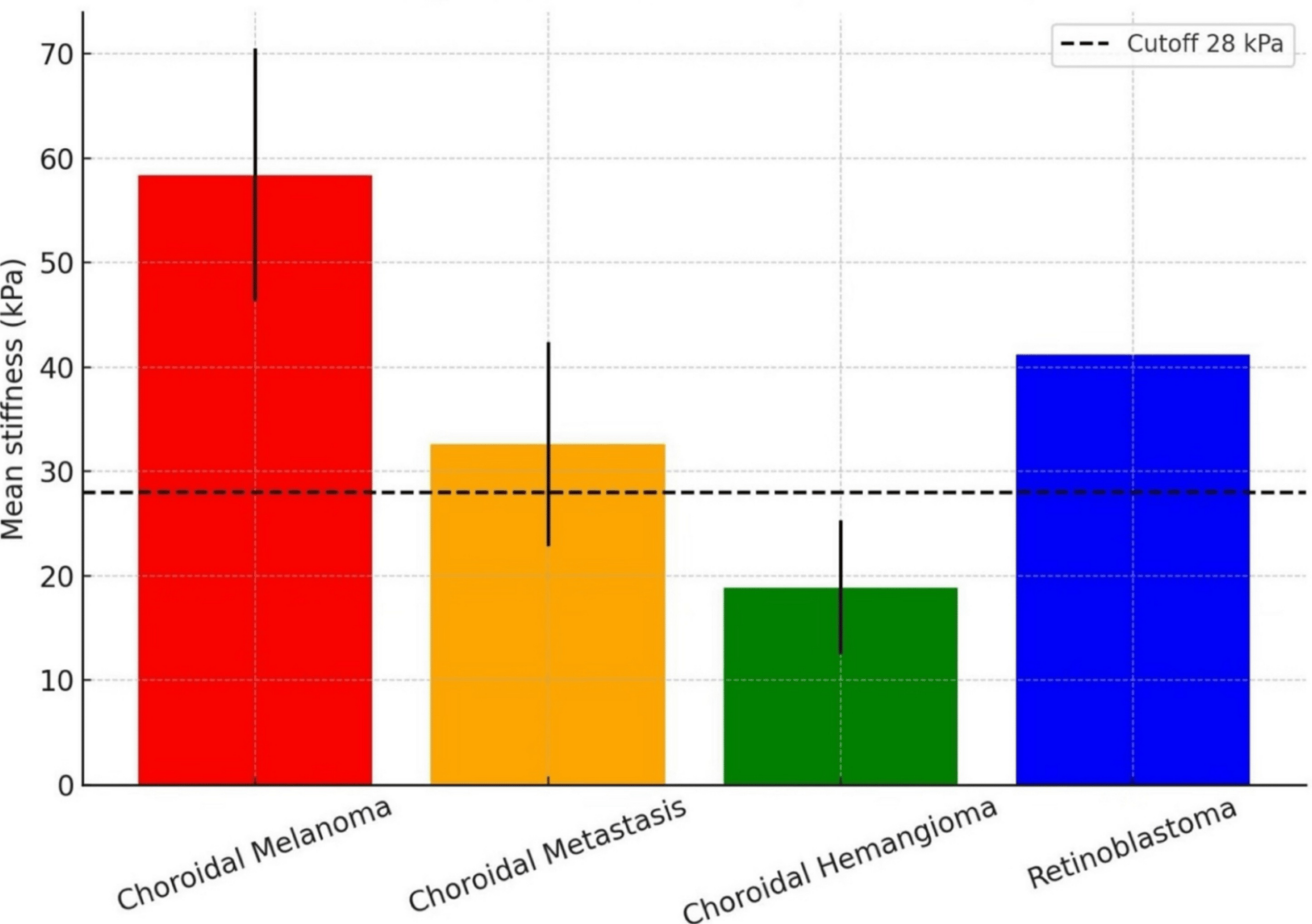
Mean tumor stiffness by lesion type

Comparative analysis revealed that benign lesions, represented by choroidal hemangiomas, had significantly lower stiffness values than malignant tumors, including choroidal melanomas and metastases (p < 0.01). A shear-wave elastography cutoff value of 28 kPa achieved a sensitivity of 91.6% and a specificity of 100% for distinguishing malignant from benign intraocular tumors (Figure 3).

## Discussion

This prospective pilot study demonstrates that the OcUsE protocol is a feasible, safe, and reproducible imaging technique for the evaluation of intraocular tumors. By providing quantitative stiffness measurements in conjunction with conventional ultrasonography, OcUsE offers complementary biomechanical information that is not captured by established ophthalmic imaging modalities. To our knowledge, this study represents one of the earliest clinical applications of combined strain and shear-wave elastography in a heterogeneous cohort of ocular neoplasms.

The consistent technical feasibility observed in this series supports the incorporation of OcUsE into clinical workflows, particularly given its operational efficiency and favorable safety profile. Rather than representing a mere extension of conventional ultrasonography, elastography introduces a functional dimension that may enhance lesion characterization without increasing procedural burden. Its performance parallels that reported in other clinical domains, where elastography has transitioned from investigational use to a complementary diagnostic tool with established clinical utility [1-3].

The variability in stiffness measurements across tumor subtypes should be interpreted within a broader biomechanical context. Increased stiffness in choroidal melanoma likely reflects intrinsic tumor architecture, including higher cellular density and stromal organization, both of which are associated with more rigid tissue properties. In contrast, the heterogeneity observed in metastatic lesions may be indicative of their diverse histologic origins and infiltrative patterns. The low stiffness values in hemangiomas reinforce the concept that vascular lesions exhibit reduced resistance to deformation, while the mixed elastographic profile in retinoblastoma suggests that internal features such as calcifications may introduce measurement variability. Collectively, these findings align with trends described in systemic oncology, where elastography captures biomechanical signatures associated with tumor biology rather than serving as a standalone diagnostic discriminator [4-7].

The proposed shear-wave elastography threshold of 28 kPa should be considered exploratory and context-dependent. Although it demonstrated favorable diagnostic performance in this cohort, its clinical applicability requires cautious interpretation, particularly in light of tumor heterogeneity and technical factors that may influence stiffness quantification. Rather than functioning as an absolute cutoff, this parameter may be more appropriately integrated into a probabilistic diagnostic framework, aiding in the stratification of lesions with indeterminate features on conventional imaging. In this regard, elastography may have its greatest impact as an adjunctive tool that refines clinical decision-making rather than replacing established modalities [8-10].

The observed concordance between elastographic findings and conventional imaging supports a multimodal diagnostic approach in ocular oncology. While B-scan ultrasonography and OCT remain fundamental for structural evaluation, elastography contributes complementary biomechanical information that may enhance lesion assessment. The MRI continues to play a central role in tumor staging and characterization, particularly in retinoblastoma and complex uveal tumors [9,4]. However, its limitations in accessibility, cost, and feasibility in certain patient populations highlight the potential role of OcUsE as a practical, real-time adjunct. Within this framework, elastography may be particularly valuable in settings requiring rapid evaluation or longitudinal monitoring, although further validation in larger cohorts is warranted to define its precise clinical role.

Reproducibility is a critical prerequisite for clinical implementation of any quantitative imaging technique. In this study, shear-wave elastography demonstrated excellent interobserver and intraobserver agreement, addressing a common concern reported in elastography applications across multiple medical fields [20-22].

Several limitations merit consideration. The pilot nature of the study and the small sample size, particularly the inclusion of a single retinoblastoma case, limit the generalizability of the findings. In addition, histopathologic confirmation was not available for all tumors, reflecting routine clinical practice in ocular oncology, where diagnosis and management are frequently guided by multimodal imaging rather than tissue biopsy [23,24]. Future studies should focus on multicenter validation, larger cohorts, histopathologic correlation where feasible, and longitudinal assessment of elastographic changes over time. Continued technological advances, including high-frequency probes optimized for ophthalmic elastography and refined biomechanical modeling, may further enhance spatial resolution and diagnostic accuracy [25,26].

## Conclusions

The OcUsE is shown to be a safe, feasible, and reproducible imaging technique for the evaluation of intraocular tumors within the context of this pilot study. Distinct and biologically plausible stiffness patterns were observed across tumor types, with choroidal melanomas demonstrating higher stiffness values, metastases exhibiting heterogeneous profiles, hemangiomas showing lower stiffness, and retinoblastoma presenting intermediate stiffness with patchy heterogeneity. The high interobserver agreement supports the technical reliability of the measurements obtained.

While the preliminary diagnostic performance findings suggest that elastography may have potential as a complementary tool for differentiating benign from malignant intraocular lesions, these results should be considered exploratory and hypothesis-generating rather than definitive. Given the limited sample size and pilot design, quantitative cutoff values and diagnostic metrics require validation in larger, prospective, multicenter cohorts before clinical integration can be considered. Further investigation is necessary to define the precise role of ocular elastography within the multimodal imaging framework of ocular oncology.
